# What Do We Know about the Role of miRNAs in Pediatric Sarcoma?

**DOI:** 10.3390/ijms160716593

**Published:** 2015-07-22

**Authors:** Lorna C. Kelly, Antonio Lázaro, Maureen J. O’Sullivan

**Affiliations:** 1 The National Children’s Research Centre, Lady’s Children’s Hospital, Crumlin, Dublin 12, Ireland; E-Mails: kellyl15@tcd.ie (L.C.K.); antonio.lazaro.cabeza@gmail.com (A.L.); 2 Histology Laboratory, Lady’s Children’s Hospital, Crumlin, Dublin 12, Ireland; 3 Trinity College, University of Dublin, Dublin 2, Ireland

**Keywords:** microRNA, regulation of gene expression, pediatric, sarcoma

## Abstract

Non-coding RNAs have received a lot of attention in recent years, with especial focus on microRNAs (miRNAs), so much so that in the just over two decades since the first miRNA, *Lin4*, was described, almost 40,000 publications about miRNAs have been generated. Less than 500 of these focus on sarcoma, and only a fraction of those on sarcomas of childhood specifically, with some of these representing observational studies and others containing functionally validated data. This is a group of cancers for which prognosis is often poor and therapeutic options limited, and it is especially in these areas that strides in understanding the role of non-coding RNAs and miRNAs in particular are to be welcomed. This review deals with the main forms of pediatric sarcoma, exploring what is known about the diagnostic and prognostic profiles of miRNAs in these tumours and where novel therapeutic options might present themselves for further exploration.

## 1. Introduction

Predicated upon the central dogma of genetics, namely that “DNA makes RNA, makes protein” [1,2], a long-held belief was that the bulk of transcriptional output of the human genome was from protein-coding mRNA, with only small amounts of non-coding RNA (ncRNA) represented. Consequently, messenger RNA (mRNA) has been the prime focus in the study of RNA species. Completion of the Human Genome Project, however, has revealed that only ~1.2% of the transcriptional output of the human genome is protein-coding, with >98% consisting of ncRNA derived from introns and exons/introns of non-protein coding genes [3]. ncRNAs are functional RNA molecules that are not translated into proteins. Some ncRNAs have been known for many years, including transfer RNA (tRNA) and ribosomal RNA (rRNA), the roles of which are well-established in protein translation. With many erstwhile “junk” RNAs now being recognized as functional, a somewhat improved understanding of ncRNAs is emerging, thanks to advances in molecular biology and improved sequencing techniques. Evidence is accruing that ncRNAs control chromosome dynamics, RNA splicing and editing, translational inhibition and mRNA destruction.

There are two main classes of ncRNA based on strand length, (which is one means of categorizing ncRNAs); those ~300 bp–100 kb in size are known as long non-coding RNAs (lncRNAs) and ncRNAs ≤ 300 bp in size are called small ncRNAs. LncRNAs are poorly conserved mRNA-like species, transcribed by RNA polymerase II, capped and poly-adenylated, but lacking an open reading frame [4,5,6]. It is estimated that there are thousands of lncRNAs within the human genome, but very little is known about the function of the vast majority of these, as only a few have been studied to date, thereby revealing roles in X-chromosome inactivation, nuclear organization and compartmentalization, as well as nuclear-cytoplasmic trafficking [7]. The complex developing landscape of lncRNAs has been the subject of multiple recent reviews [8,9]. Small ncRNAs include tRNAs, rRNAs, small nuclear RNAs (snRNAs), RNase P, RNase MRP, telomerase RNAs, piwi-interacting RNAs (piRNAs), small nucleolar RNAs (snoRNAs), small interfering RNAs (siRNAs) and microRNAs (miRNAs). Of these, miRNAs are the most extensively studied subclass and will form the subject of this review.

MicroRNAs (miRNAs) are evolutionarily conserved, small, single-stranded ncRNAs, typically 18–25 nucleotides (nt) long [10,11,12,13]. The main function of miRNAs is in the negative regulation of gene expression by interfering with protein expression either through inhibition of translation, or through mRNA degradation [13], with the latter representing the predominant mechanism of action [14]. In some instances miRNAs have the ability to *activate* mRNA translation [15]. miRNAs have been shown to play major roles in both animal and plant development and estimates suggest that they control 30%–50% of all human genes. The first miRNA, *Lin4*, was identified in the early 1990s in *Caenorhabditis elegans* (*C. elegans*) and is involved in early larval transition [16,17]. It has anti-sense complementarity to the 3′ UTR of *lin14* such that *lin4* binding to *lin14* leads to reduced protein without affecting mRNA expression [16,17]. Until 2000, upon the discovery in *C. elegans* of lethal-7 (let-7) similarly important in larval transition [18], *Lin4* was thought to be the only such ncRNA in existence. Let-7 homologues were soon identified in *Drosophila melanogaster* and humans [10,11,12,19] and by 2001 over one hundred short temporal RNAs (stRNAs), as they were briefly known, had been identified. While most of these were of similar size and function and underwent similar processing, the majority were not in fact expressed at distinct stages of development but more likely in specific cell types. In 2001, they were designated microRNAs (miRNAs) [10,11,12].

miRNA biogenesis and processing may begin in either of two ways: the canonical pathway or the mirtron pathway [10,20,21]. In the canonical pathway, miRNAs are transcribed in the nucleus as large primary transcripts from either distinct miRNA encoding genes or from genes located within protein coding genes [10]. These primary transcripts, usually >1 kb in length, are known as primary-miRNAs (pri-miRNAs) [22]. They contain a cap structure at the 5′ end and a 3′ poly-adenylated tail [23,24]. These large pri-miRNA are processed and cleaved into a 60–70 nt stem loop precursor-miRNA (pre-miRNA), by Drosha and a number of its co-factors including DGCR8 proteins [22,25,26,27]. Drosha is a double-stranded, RNA-specific, highly conserved RNaseIII endonuclease, which cleaves both strands of the pri-miRNA at the base of the stem-loop structure [25,28]. Some miRNAs are produced through the alternative mirtron pathway from very short introns of different genes transcribed in the nucleus [20,29,30]. These miRNAs are produced as 60–70 nt pre-miRNAs, as a result of debranching and splicing of pre-mirtrons, therefore by-passing the need for processing and cleavage by Drosha and its co-factors [21].

**Figure 1:**
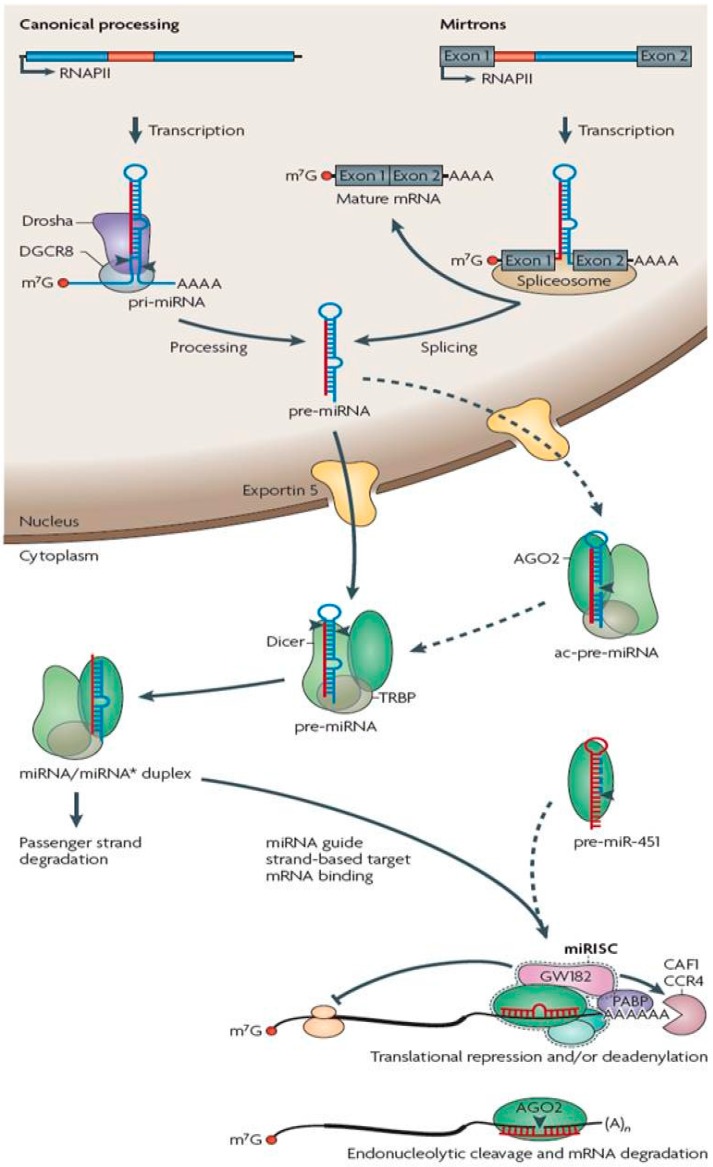
miRNA biogenesis. miRNAs are transcribed as long primary miRNAs (pri-miRNAs) in the nucleus by the canonical processing or from mirtrons. In the canonical processing pathway, pri-miRNAs are cleaved into 60–70 nt precursor miRNAs by the RNaseIII endonuclease DROSHA and transported to the cytoplasm by RAN-GTP dependent exportin-5, or in the mirtron pathways, pre-miRNAs result from debranching and splicing of pre-mirtons. In the cytoplasm, the pre-miRNA is cleaved by DICER into a short miRNA/miRNA* duplex and the mature miRNA can then be loaded into the miRISC and exert its function on its target mRNA, by target degradation or repression (Adapted from Krol *et al.* 2010) [31].

Whether the pre-miRNA is produced by the canonical or mirtron pathway, the mechanism of mature miRNA production remains the same [31]. The 60–70 nt pre-miRNA is actively transported from the nucleus into the cytoplasm by its interaction with the RAN-GTP dependent protein Exportin 5 [22,32,33,34] and once in the cytoplasm, is cleaved by Dicer, also an RNaseIII endonuclease, and its cofactors, TRBP and PACT, to produce a short (~20 bp) imperfect double stranded miRNA/miRNA* complex [22,35,36,37]. In mammals, the Argonaute (AGO) 2 protein, with strong RNaseIII-like endonuclease activity, can also support Dicer [38]. The miRNA/miRNA* duplex contains the mature miRNA, or “guide strand”, and a “passenger strand” (miRNA*). The duplex is unwound and the guide strand incorporated into the miRNA-induced silencing complex, miRISC, of which the Argonaute proteins (AGO 1–4) are core components [37,39,40]. Once loaded into the miRISC, the mature miRNA can exert its function on its target mRNAs (Figure 1). Mature miRNAs can reside on either strand of the miRNA/miRNA* duplex, but mostly derive from the less stably paired 5′ end [41,42,43,44] reflecting the ease of unwinding from the 5′ end of the duplex. The released passenger strand (miRNA*) is usually degraded, but in cases where the components of the miRNA/miRNA* duplex have similar stability, each strand may be loaded into the miRISC equally [41,42].

The roles of miRNAs in development and cancer are now well-established, and herein we will discuss what is currently known about the role of miRNAs in pediatric sarcomas in terms of cancer biology and also diagnostic, prognostic and therapy-predictive value.

## 2. Osteosarcoma

### 2.1. Clinical Background and Research Challenges

Osteosarcoma is the most common human malignant primary bone tumour, typically arising within the long bones in patients in their second and third decades of life, with a smaller second peak in later adulthood. Osteogenic sarcoma, as the name implies, is defined by the production of osteoid matrix by malignant cells, which ultimately destroy the native bone, often produce an associated soft tissue mass and have a great propensity for lung metastases. Treatment with surgery alone historically resulted in a less than 20% 5-year survival, and while the introduction of chemotherapy has improved outcomes somewhat, despite considerable advances in the outcomes for most other pediatric cancers, there has been no significant improvement for osteosarcoma in over thirty years, with a currently still very disappointing 60% 5-year overall survival rate [45,46]. Outcomes for patients with metastatic or recurrent disease are particularly dismal, and the molecular mechanisms underlying the development of metastasis and drug resistance are poorly understood. This lack of real progress in understanding osteosarcoma is probably multifactorial. Not only does osteosarcoma constitute a number of histological subtypes and potentially display remarkable genomic instability [47], creating considerable challenges in the investigation of driver mutations in this tumour type, but working with bony tissue adds its own technical issues. It is reassuring in this context that miRNA profile studies have consistently produced clustering of osteosarcomas separate from other sarcoma or healthy tissue types [48,49,50]. The seemingly quite diverse results emerging from miRNA studies of osteosarcoma relate to the fact that certain of these studies compare miRNA profiles of tumour samples and/or cell lines to osteoblasts differentiated in culture conditions, while others have focussed more on the osteosarcoma-specific miRNA profiles or indeed “back-tracked” from gene expression observations in osteosarcoma to potentially accountable miRNAs. Recurring themes do however emerge from these investigations.

### 2.2. Syndromic Associations and Genetic Insights

Certain germline predispositions to osteosarcoma have long been known, including mutations of *RB1* gene in hereditary retinoblastoma, *TP53* as in the Li–Fraumeni syndrome, or various of the DNA helicases [51,52,53]. Further oncogenic mechanisms may involve interacting genes, or indeed miRNAs affecting these key pathways. A comprehensive analysis of paired osteosarcoma and constitutional DNA by complementary genomic approaches recently showed that while *TP53* mutations were identified in 22% osteosarcomas, complete P53 pathway inactivation was in fact seen in 75% cases, and in about a third of cases, multiple mechanisms of inactivation of P53 were observed [47].

### 2.3. miRNAs Involved in Major Pathways

In terms of miRNAs, evidence has emerged that certain miRNAs, notably *miR-*34, are involved in the P53 pathway, such that in the cancer context, the *miR-*34 family promote cell growth arrest and cell death, and indeed *miR-*34 may show reduced expression in osteosarcoma [54] (Table 1). *miR-*31 targets the cell cycle regulator E2F2 and, upon over-expression, inhibits proliferation of osteosarcoma cell lines; loss of *miR-*31 produces defects in the TP53 pathway [55]. TP53 can induce expression of *miR-*215 and *miR-*192, resulting in p21 expression. *miR-*192 reduces osteosarcoma cell colony formation, corroborating its role as a growth/tumour suppressor in osteosarcoma [56] while *miR-*215 similarly reduces cell proliferation.

Apart from the commonly affected TP53 and RB1 pathways in osteosarcoma, Perry *et al.* [47] reported genomic alterations in the PI3K/mTOR pathway in a full 24% of cases in their cohort, highlighting potential therapeutic targetability thereof. In relation to miRNAs in this context, *miR-*223 is thought to have a tumour-suppressing role through the PI3K/Akt/mTOR pathway in osteosarcoma. Indeed, combined low *miR-*223 with high target mRNA *ECT2* expression in osteosarcoma was significantly associated with tumour grade, chemo-resistance, development of metastases and tumour recurrence [57], as was in that same cohort, the combined low *miR-*183 with high target mRNA *EZRIN* expression. Additional tumour suppressor roles have been described for *miR-*199a-3p which inhibits cell growth and migration and is thought to target MET, mTOR and STAT3 [58]. STAT3 is similarly targeted by *miR-*125b with a feedback loop whereby STAT3 binds the promoter leading to transcription of *miR-*125b [59] (Table 1).

Zhao *et al.* [60] found significant down-regulation of *miR-*133a/b in osteosarcoma samples compared with normal controls and noted that over-expression of *miR-*133b, particularly, significantly reduced cellular migration and invasion. Predicted target pathways of *miR-*133b, including Bcl2L2, Mcl1, IGF1R, MET, pAkt, PTEN and FAK, all showed reduced expression of proteins within these pathways in osteosarcoma cell lines upon over-expression of *miR-*133b. Novello *et al.* had previously identified a role for down-regulation of *miR-*133b in cell proliferation in osteosarcoma [61].

### 2.4. miRNAs and Metastasis

As Fas ligand is expressed constitutively in only 4 human tissues including the lung, where osteosarcoma preferentially metastasises, Huang *et al.* focussed on identifying miRNAs that might target Fas. Increased expression of *miR-*20a, a member of the *miR-*17-92 cluster, combined with decreased Fas expression was associated with reduced FasL-induced apoptosis and cell cytotoxicity and contributed to the development of lung metastases in osteosarcoma. miRNAs at the 14q32 locus are significantly down-regulated in osteosarcoma, and a subset of these act cooperatively to down-regulate MYC, which in turn down-regulates the *miR-*17-92 cluster [62].

### 2.5. miRNAs, Chemotherapy and Outcomes

Separate studies focussing on individual miRNAs over-expressed in osteosarcoma suggested higher *miR-*214 [63] and *miR-*210 expression [64] were predictive of poor pre-operative chemo-responsiveness, as well as shorter overall and event-free survival for high *miR-*214-expressing tumours, and increased metastatic risk as well as reduced overall and progression-free survival for higher *miR-*210 expressing tumours. Pre-treatment surgical samples [65] showed higher *miR-*27a and *miR-*181c levels in cases that developed metastases, while higher *miR-*451 and *miR-*15b correlated with a positive subsequent response to chemotherapy. *miR-*15 and *miR-*16 target Bcl-2 which might contribute to the improved chemo-responsiveness of cases with higher *miR-*15b. Zhang *et al.* also suggest Bcl-2 as a target of *miR-*143, which functions as a tumour suppressor in osteosarcoma [66].

Low expression of *TWIST* mRNA has been associated with chemo-resistance in osteosarcoma and in an effort to identify miRNAs involved, Zhou demonstrated consistently lower *TWIST* and higher *miR-*33a levels in osteosarcomas chemo-resistant to cisplatin [67]. From the work of Braun *et al.* [56], *miR-*215 was associated with chemo-resistance to methotrexate. High *miR-*140 expression was identified in a screen for miRNAs associated with osteosarcoma chemo-resistance including to doxorubicin, cisplatin and ifosfamide [68], while a 5-miRNA signature consisting of *miR-*92a, *miR-*99b, *miR-*193a-5p and *miR-*422a over-expression and *miR-*132 down-regulation was associated with resistance to ifosfamide in human and rat osteosarcoma [69]. These miRNAs target the TGFb, Wnt and MAPK pathways, similarly identified as targets of 38 miRNAs differentially expressed in osteosarcoma, to include Notch, RAS/MAPK, Wnt and Jun/Fos pathways [70] (Table 1).

## 3. Ewing Sarcoma (ES)

### 3.1. History, Clinical Background, Tumour Genetics

Ewing sarcoma is the second most common bone sarcoma of children and young adults, but may arise also in soft tissue or indeed visceral locations and indeed rarely in older adults. The Ewing sarcoma family of tumors (ESFT) includes peripheral primitive neuroectodermal tumor (pPNET) and Askin tumor (Ewing sarcoma of the chest wall). Early diagnosis with localised disease has ~70% 5-year survival rate, however 20%–30% of cases present with metastasis, and for these, prognosis is poor. Survival with relapsed ESFT is <30%. The defining feature of ESFTs is a chromosomal translocation that fuses EWSR1 on chromosome 22q12 to a member of the ETS family of transcription factors. The most common translocation, found in 85% of cases is a t(11;22)(q24;q12) translocation, which fuses EWSR1 to Fli1 on chromosome 11. In a further 10% of cases EWSR1 is fused to ERG1 on chromosome 21 resulting from the chromosomal translocation t(21;22)(q22;q12). Rarely, EWSR1 can fuse with ETV1, ETV4, FEV, NSG or NFATc2 [71]. In further very rare cases, FUS/TLS gene, rather than the related EWSR1, can fuse to ERG1 or FEV, or indeed EWSR1 fuses to a non-ETS gene [72]. EWSR1-Fli1 is believed to act as an aberrant transcription factor that transforms target cells by deregulating their gene expression program. It is thought to regulate ~1000 genes, with around 80% of these down-regulated [73,74]. The fusion product activates and represses pathways important for tumorigenesis, including the IGF1R pathway, Cyclin D1 and p21 regulation of cell cycle and TGFβ signalling [75]. EWSR1-Fli1 was classified as an oncogene due to its ability to transform NIH3T3 cells; however, expression in most normal primary human cells induces cell death [76,77].

### 3.2. Overexpressed miR Clusters

Three paralogous miR clusters—*miR-*17-92a, *miR-*106b-25 and *miR-*106a-363—known to be highly conserved across species and believed to have arisen from genetic duplications are up-regulated in Ewing sarcoma [78] (Table 1). Blockade of miR clusters has been shown to be far more effective than blockade of miR families [78]. Several members of these clusters are associated with poor prognosis in Ewing sarcoma [79]. Blockade of the *miR-*106a-363 cluster is most potent in inhibiting colony formation by ES cell lines. This inhibition of *miR-*106a-363 is associated with the over-expression of *miR-*15a with a partial rescue of the 106a-363 cluster expression-induced growth inhibition of ES cell lines by this up-regulation of *miR-*15a. Based on these data, blockade of *miR-*106a-363 cluster and restoration of miR15a both offer promise as therapeutic options in Ewing sarcoma [78].

### 3.3. EWSR1-Fli1 and miRNA Targets

De Vito *et al.* reported that compared with human mesenchymal stem cells, Ewing sarcoma cell lines showed repression of the let-7 miR family, including *miR-*100, *miR-*125b and *miR-*31, as well as over-expression of the *miR-*17-92 cluster and its paralogues *miR-*106a/b (similar to Dylla’s observations above). De Vito demonstrated direct binding of the EWSR1-Fli1 fusion protein to the promoter region of Let-7a, a known growth/tumor suppressor with important roles in differentiation, and this resulted in repression of pri-let-7a-a and mature let-7a [80]. ESFT tumour cell growth reduction resulting from over-expression of let-7a was shown to be mediated through HMGA2 repression, a target of let-7a along with IGF2BP1 and Lin28 [80]. Zhang *et al.* reported similar growth effects with ectopic expression of let-7 inhibiting cell proliferation, migration and invasion, arresting cell cycle progression and inducing apoptosis [81] (Table 1).

McKinsey *et al.* investigated miRNAs up-regulated following shRNA silencing of EWSR1-Fli1 and identified, amongst the top-ranking miRNAs, seven which target the IGF pathway specifically [82]. All of these were subsequently shown to have growth suppressive effects. Considerable overlaps with the findings of De Vito *et al.* exist in the miRNAs reported as altered by McKinsey, and evidence of direct targeting of these miRNAs by EWSR1-Fli1 is reported. The repression of IGF1R and mTOR by *miR-*100 and of IGF1a by *miR-*27a suggest post-transcriptional de-repression of the IGF pathway as an oncogenic mechanism in Ewing sarcoma, mediated by EWSR1-Fli1.

The two key biomarkers of, and oncogenic contributors to, Ewing sarcoma, namely EWSR1-Fli1 fusion protein and CD99 expression are linked through *miR-*30a-5p, which was shown by luciferase assay to bind CD99 [83]. While evidence of direct binding of EWSR1-Fli1 to *miR-*30a-5p is lacking, over-expression of *miR-*30a-5p leads to reduction of cell proliferation and invasion as well as reduced CD99 expression in multiple Ewing sarcoma cell lines [83].

Candidate tumour suppressor miRNAs normally repressed by EWSR1-Fli1, included *miR-*22 [82], shown to have tumour suppressor properties in other cancers [84,85]. Ectopic expression of *miR-*22 in ES cell lines (A673, SK-ES-1 & SK-N-MC) inhibited colony formation in all three cell lines tested compared with controls [86]. Of four predicted target genes involved in chromatin remodelling, *miR-*22 over-expression led to a 20% decrease in the histone demethylase *KDM3A* mRNA and silencing of KDM3A caused a robust inhibition of colony formation [86]. KDM3A is up-regulated in Ewing sarcoma and this is at least in part as a result of EWSR1-Fli1 expression, which appears to act by repressing *miR-*22 and in turn de-repressing KDM3A [86]. KDM3A up-regulates a number of oncogenes including Ets1, an effector of tyrosine kinase signalling and a known positive regulator of Cyclin D1 (Table 1).

### 3.4. miRNAs and Embryonic Stem Cell Genes

The cell of origin of Ewing sarcoma remains unclear, but studies have suggested human mesenchymal progenitor cells (hMPCs) as a likely candidate cell of origin for Ewing Sarcoma [87,88]. *miR-*145 is a target of EWSR1-Fli1 fusion protein [89,90] and shows low/absent expression in Ewing sarcoma cells compared with human mesenchymal stem cells. *miR-*145 in turn directly targets OCT4 and SOX2, the expression of which can, along with NANOG, (a further embryonic stem cell gene), be induced by EWSR1-Fli1 in pediatric human mesenchymal stem cells, but not the adult hMSC counterpart [90]. *miR-*145 directly binds Fli1 3′ UTR and it can reduce the EWSR1-Fli1 protein level without affecting mRNA levels [89,90]. Thus, a mutually repressive feedback loop of EWSR1-Fli1 and *miR-*145 has been uncovered, with their common targets gene SOX2 and miRNA-145 themselves key players in stem cell differentiation [91] and tumorigenicity [90].

### 3.5. Copy Number Alterations with Progression and miRNA Expression

In a comparison of aCGH and miRNA expression data from Ewing sarcoma xenografts over many passages, Mosakhani *et al.* identified copy number losses most commonly at chromosome 9q21.3 and 16q [92]. Frequent gains involved whole chromosomes 8 and 15 together with gain at 17q21.32-qter, and gain at 1q21.1-qter was also observed. miRNAs showing significant over-expression compared with hMSCs were *miR-*106b, *miR-*93, *miR-*181b, *miR-*101 and *miR-*30b, while *miR-*145, *miR-*193a-3p, *miR-*100, *miR-*22, *miR-*21 and *miR-*574-3p showed significantly lower expression [92]. Many of these overlap with the findings of DeVito and Dylla above. Twelve miRNAs were expressed only in primary xenografts and not in lung metastases and 11 of these were expressed in primary and control xenografts, but not metastases; these include *miR-*214*, *miR-*154*, *miR-*337-3p, *miR-*369-5p, *miR-*409-5p, *miR-*411, *miR-*485-3p, *miR-*478qa and *miR-*770-5p [92]. Eighteen miRNAs were expressed only in lung metastases and not in primary xenografts. These investigators furthermore reported 41 miRNAs differentially expressed in lung metastases, 8 of which are from the 14q32 imprinted region and these all showed reduced expression in metastases compared with xenografts, suggesting a role in tumour progression. As no copy number alterations in this 14q32 region were reported by the investigators, the possibility exists that the reduced expression of the relevant miRNAs results from promoter methylation, a mechanism previously invoked by several investigators for the loss of tumour-suppressive effect in the context of gastrointestinal stromal tumour (GIST) [93,94,95].

### 3.6. miRNAs, Chemo-Responsiveness and Outcomes

Nakatani *et al.* carried out miRNA expression profiling on 34 ES biopsies (21 early relapse and 13 no relapse) [79]. Analysis revealed 15 miRNAs significantly associated with event-free survival (EFS) and 32 miRNAs significantly associated with overall survival (OS), and of these, five were significantly associated with survival prediction (*miR-*34a, *miR-*23a, *miR-*92, *miR-*490-3p and *miR-*130b) [79]. Multivariate analysis confirmed that these 5 miRNAs are independent predictors of patient outcome when age, sex, and tumour location are introduced as covariates. In a larger cohort of Ewing sarcomas only *miR-*34a and *miR-*490-3p achieved sufficient power to predict prognosis. *miR-*34a and *miR-*490-3p were both significantly lower in patients who relapsed compared to patients with no evidence of disease. Higher expression of *miR-*34a was associated with a better outcome when either EFS or OS were considered. *miR-*490-3p expression only correlated with OS. Patients with the highest expression of *miR-*34a did not experience any adverse events, whereas patients with the lowest expression mostly recurred within two years [79]. In Ewing sarcoma cell lines, *miR-*34a expression was found to be inversely associated with anchorage-independent colony formation. *miR-*34a expression is regulated by p53 and so the relationship between p53 and *miR-*34a was investigated. ES cells with p53 mutations (SK-N-MC, SKES1, TC71 & IOR/CAR) have lower expression levels of *miR-*34a compared to wild-type p53 ES cells (WE-68, IOR/RCH and LAP-53) and inhibition of *miR-*34a in p53 WT LAP-35 cells significantly increased anchorage-independent growth, while restoration of *miR-*34a in p53 mutant cells decreased anchorage independent growth. Ectopic expression of *miR-*34a also sensitised ES cells to vincristine and doxorubicin, lowering the IC_50_ in the cells, raising consideration of *miR-*34a as a potential therapeutic agent in Ewing sarcoma [79].

While outcomes for patients presenting with localised Ewing sarcoma have improved considerably in recent decades, this is not the case for metastatic presentations or recurrent disease [96,97]. Histological evaluation of chemo-response is central to prognostication for Ewing sarcoma, and as many chemotherapeutic agents are DNA-damaging, Robin *et al.* elected to study EYA family proteins in Ewing sarcoma [98]. EYA proteins are involved in DNA repair and qRT-PCR evaluation of hMSC and Ewing sarcoma cell lines revealed that only EYA3 is consistently up-regulated in Ewing sarcoma. EWSR1-Fli1 knock-down led to reduced *EYA3* mRNA levels, and despite prior suggestions to the contrary, these investigators found no evidence of direct binding of the fusion protein to the EYA3 promoter and therefore opted to investigate the role of miRNAs in this context [98]. Analysis of the EYA3 3′ UTR with miRNA target prediction software revealed three miRNAs predicted to bind to EYA3: *miR-*145, *miR-*28-5p and *miR-*708. Of these, a role for *miR-*708 was most compelling, with proven direct targeting of EYA3 by miRNA-708 [98], over-expression of *miR-*708 leading to reduced EYA3 expression, an inverse correlation of *miR-*708 and EYA3 levels in primary tumours, as well as increased ES cell sensitization to etoposide by either EYA3 silencing or *miR-*708 expression, mediated by increased apoptosis. Moreover, a clear trend emerged, whereby patients whose tumours had low levels of *miR-*708 and high EYA3 had worse three-year relapse-free survival rates [98]. Based on these findings, Robin *et al.* devised a model whereby *miR-*708 repression by EWSR1-Fli1 led in turn to de-repression of EYA3 resulting in increased DNA repair capacity and reduced chemo-response.

Further work on the role of miRNAs in chemo-sensitivity of Ewing sarcoma came from Lida *et al.* Profiling of 46 miRNAs in resistant and parental Ewing sarcoma cell lines showed *miR-*125 was the most highly up-regulated miRNA in the doxorubicin-resistant cell lines [99]. Silencing of *miR-*125b in doxorubicin-resistant ES cells (VH-64/ADR & WE-68/ADR) significantly enhanced cell death by increasing sensitivity to doxorubicin, and this increased sensitivity to doxorubicin was also seen in the parental ES cells and in additional ES cell lines (RD-ES, SK-N-MC and TC-71 cells). Down-regulation of *miR-*125b in doxorubicin-resistant cells increased their sensitivity to vincristine and etoposide, while over-expression of *miR-*125b in the parental cells reduced cytotoxicity; knockdown of *miR-*125b enhanced the sensitivity of both cell type to mafosfamide also [99]. These findings suggest that *miR-*125b enhances chemo-resistance to multiple drugs used in the standard regimens for ES. The mechanism here may be through suppression of apoptotic mediators including p53 and Bak [99] and over-expression of miRNA-125b furthermore inhibited cell migration and invasion in tandem with down-regulation of PI3K/AKT/mTOR signalling by targeting PIK3CD (Table 1).

## 4. Rhabdomyosarcomas (RMS)

### 4.1. Clinical Background and Tumour Genetics

Rhabdomyosarcoma is the most common soft tissue sarcoma in childhood and is classified into two major histological subtypes, namely embryonal rhabdomyosarcomas (ERMS) and alveolar rhabdomyosarcoma (ARMS). Of these, ARMS has a more primitive phenotype with round/ovoid largely undifferentiated cells that have high nuclear:cytoplasmic ratios, while a greater degree of differentiation characterises the tumour cells of ERMS. ERMS is associated with aberrations including loss of heterozygosity and loss of imprinting at 11p15.5 as well as gains of chromosomes 2, 8, 12 and 13. In contrast, alveolar rhabdomyosarcomas (ARMS) are associated with t(2;13) or less commonly t(1;13) chromosomal translocations producing a PAX3-FOXO1 or PAX7-FOXO1 fusion gene respectively. Rhabdomyosarcoma represents a tumour arising from abortive skeletal muscle differentiation with immunohistochemical features to support this.

Of the pediatric sarcomas, embryonal rhabdomyosarcoma, notably ERMS of the uterine cervix, must be considered a potential element of germline *DICER1* mutation-associated syndromes, which incorporate a range of dysontogenetic manifestations and embryonal cancers [100,101,102]. *DICER1* is a double-stranded RNA-specific endoribonuclease, essential to the processing of miRNAs (Figure 1). ERMS is of course also a potential manifestation of the Li–Fraumeni syndrome [51] and young presentation (<2 years old), particularly with ERMS, should prompt an in-depth investigation of family cancer history and germline TP53 mutational analysis.

### 4.2. MyomiRs

Several miRNAs exhibit exquisite tissue specificity, notably *miR-*1-1, *miR-*1-2, *miR-*133a-1 and *miR-*133a-2, which are almost exclusively expressed in striated (cardiac and skeletal) muscle. Additionally, *miR-*133b and *miR-*206 are exclusively expressed in skeletal muscle and have identical sequences to *miR-*133a and *miR-*1. *miR-*1, *miR-*133a, *miR-*133b and *miR-*206 are collectively referred to as myomiRs [103]. All 4 myomiRs are expressed at significantly lower levels in RMS compared with skeletal muscle samples [103]. The expression of *miR-*206 was found to be significantly inversely correlated with overall survival, while lower *miR-*206 expression was significantly associated with shorter survival in ERMS and ARMS fusion-gene negative cases. This relationship was not found in ARMS fusion positive cases [103] (Table 1).

Indeed, analysis showed that *miR-*1, *miR-*133a, *miR-*133b and *miR-*206 were useful serum biomarkers for distinguishing RMS patients from non-RMS patients, with *miR-*206 showing the highest specificity [104]. The expression level of each myomiR was decreased in serum after treatment of RMS and therefore use as biomarkers for relapse might be considered [104]. miRNA arrays were run on embryonal (RD) and alveolar (Rh30) rhabdomyosarcoma cell lines in parallel with skeletal muscle samples. The expression of myomiRs was found to be drastically reduced in the RMS cells compared with normal skeletal muscle. The most dramatic differences in miRNA expression were for *miR-*1 and *miR-*133a [105]. In RD cells ectopic expression of both *miR-*1 and *miR-*133a inhibited cell growth with *miR-*1 acting mainly through a specific G1-S phase arrest [103]. Over-expression of *miR-*1 was found to play an instructive role in RMS cells and promoted differentiation with levels of myogenin and MyL1 five times higher in *miR-*1 transfected cells compared to controls [103]. The different sensitivities of RD and Rh30 cells to these miRNAs could be due to desensitisation of Rh30 cells to *miR-*1/*miR-*133a or the PAX3-FOXO1 fusion overriding the growth inhibitory effect of *miR-*1/*miR-*133a. *miR-*1 targets HDAC4 [106], which represses myogenin and MEF2C, which synergistically transactivate and up-regulate each other’s expression. Therefore, HDAC4 is a potential mediator here in *miR-*1-induced myogenic differentiation.

In control muscle samples the absolute level of *miR-*1 was over 60 times that of *miR-*206, and in primary RMS samples, *miR-*1 levels were absent or in line with *miR-*206 levels [107]. Neither mutations of the MyoD1 or myogenin binding sites upstream of miRNA 1/206 nor evidence of hypermethylation of the miR promoters was apparent. Constitutive re-expression of *miR-*1 or *miR-*206 in RMS cells caused a ~50% reduction in colony formation in soft agar [107]. The expression of *miR-*206 also led to a shift in the global gene expression profile to myogenic differentiation [107]. Re-expression of *miR-*206 led to loss of MET expression, marked reduction in cell cycle genes, including CDK2, CDK4, E2F1, E2F3 and pRB, all important in G0/G1 progression and G1-S phase transition, and massive apoptosis plus a switch to differentiation rather than proliferation [107], as also described by Yan *et al.* [108] and Li *et al.* [105] who investigated further targets of the myomiRs 1/206, which are significantly down-regulated in RMS. Both miRNAs share seed sequences, potentially therefore having the same targets. The 3′ UTR of PAX3 has two potential binding sites for miRNA 1/206 and binding was proven by luciferase assay [105]. In ERMS, over-expression of miRNA 1/206 led to repression of PAX3 while in ARMS this effect was not observed. Notably the oncogenic fusion PAX3-FOXO1 lacks the PAX3 3′ UTR binding site and thereby appears to escape this regulation by miRNAs. CCND2 is a further target of *miR-*1/206 over-expression, and in ERMS (JR1) and ARMS (Rh30) cell lines, their low levels of expression are associated with stabilisation of PAX3 and CCND2 [105] (Table 1).

### 4.3. miRNAs and Myogenic Differentiation

*miR-*203 is down-regulated in rhabdomyosarcoma compared with normal skeletal muscle samples [109]. This repression is due to promoter hypermethylation in cell lines and tumor tissue samples. 5-aza-dC treatment of Rh30 and RD cells re-activated *miR-*203 expression and slowed down cell growth, as did ectopic expression of *miR-*203 in Rh30 and RD cells [109], similar to re-expression of *miR-*1 and *miR-*206 [103,107]. *miR-*203 re-expression promoted terminal myogenic differentiation. Re-expression of *miR-*203 targets p63, which in turn targets JAGGED1 in the NOTCH pathway, and also LIFR in the JAK1/STAT1 pathway, and in combination, these lead to decreased proliferation, migration and invasion and increased differentiation of cells [109]. *In vivo* in xenograft mice injected with RD cells, the injection of *miR-*203 following tumour formation efficiently reduced tumour growth; RT-qPCR confirmed the presence of *miR-*203 in these tumours [109]. BAF53a is a member of the SWI/SNF chromatin remodelling complex and is down-regulated concomitantly with myogenic differentiation [107]. In RMS, forced expression of *miR-*206 leads to down-regulation of BAF53a [107]. In RMS tumour samples, BAF53a was over-expressed about five-fold compared with normal muscle tissues or with proliferating normal human myoblasts [110]. A hallmark feature of rhabdomyosarcoma is the expression of muscle marker MyoD1, but despite its high expression in rhabdomyosarcoma, it is non-functional and consequently myogenesis is arrested in this context. BAF53a is a direct target of *miR-*206 and BAF53a deficient in the 3′ UTR binding sequence is resistant to differentiation induced by over-expression of the muscle-specific *miR-*206 [110]. Sustained expression of BAF53a interferes with myogenic differentiation, while silencing BAF53a in RMS cells increases expression of myogenic markers and inhibits proliferation and anchorage-independent growth [110]. Thus silencing of BAF53a appears to have a prominent role in the transition from growth to myogenic differentiation. BAF53a is one of the subunits that undergoes isoform exchange during neural differentiation from the np to pBAF complex. BAF60b and 47 are additional targets of *miR-*1/206 and their over-expression similarly has a negative effect on myoblast differentiation. BAF60c is important in the recruitment of further subunits to the myogenic SWI/SNF complex during striated muscle differentiation.

*miR-*26a, a key myogenic regulator of murine C2C12 myoblasts, was consistently down-regulated in RMS cell lines and tumour tissue samples compared with differentiating rhabdomyoblasts, and a validated target of *miR-*26a, namely the Polycomb Repressor Complex 2 (PRC2) protein Ezh2, was diametrically overexpressed [111]. Similarly, *miR-*214 is important in C2C12 myoblastic differentiation and forms a negative feedback loop with Ezh2 that, through its chromatin remodelling effect, controls not only *miR-*214 expression but also that of myogenic transcription factors MyoD1 and Myogenin [112]. *miR-*214 is markedly down-regulated in RMS and, upon reintroduction into the RMS cell line, inhibits cell growth, induces myogenic differentiation and apoptosis. N-Ras is a conserved target of *miR-*214 and its expression is diametrically linked with *miR-*214 [113].

TGFβ1 is a potent inhibitor of myogenic differentiation and as rhabdomyosarcoma essentially is a tumour of failed myogenic differentiation, Sun *et al.* [114] investigated miRNAs regulating TGFβ1 and found that amongst these, miRNA 450b-5p was regulated through SMADs signalling. siRNA knock-down of TGFβ1 led to increased levels of *miR-*450b-5p. Over-expression of *miR-*450b-5p was associated with repression of ENOX2 and PAX9 as the only targets in RMS suppressed by *miR-*450b-5p, while also showing increased expression on knock-down of *miR-*450b-5p, in RMS cells [114]. Moreover, progression of myogenic differentiation and increase in apoptosis were observed as results of knock-down of TGFβ1. Thus, these data show that miRNA 450b-5p is a tumour suppressor through its inhibition of oncogenes in RMS [114] (Table 1).

Wang investigated the NFKb which similarly to TGFβ1 is known to inhibit myogenesis through YY1 [115]. During normal myogenesis, there is de-repression of *miR-*29 due to down-regulation of its repressors NFKb and YY1, permitting increased targeting of YY1 by *miR-*29, thereby facilitating myogenic differentiation. In rhabdomyosarcoma on the other hand, there is epigenetic silencing of miRNA29 through activation of the NFKB-YY1 pathway and as a result there is reduced silencing of YY1 by miRNA 29, and in the context of higher YY1, myogenic differentiation does not progress [116].

### 4.4. miRNAs and Amplification 13q31

Amplification of the 13q31 chromosomal region in ARMS is seen in up to 23% ARMS cases and is notably associated with PAX7-FOXO1 rearrangement [117]. Located within the minimum common region of amplification 13q31 are 2 genes including MIR17HG, which contains the *miR-*17-92 cluster, and pseudogene LC390419. Amplification 13q31 was coupled with the over-expression of 5/6 mature miRNAs of the 17-92 cluster (*miR-*17, *miR-*19a, *miR-*19b, *miR-*20a and *miR-*92a) [117]. A significantly worse outcome was associated with increased expression of the five miRNAs (*miR-*17, *miR-*19a, *miR-*19b, *miR-*20a and *miR-*92a) in 13q31-amplified cases compared with non-amplified cases, and there was also an improved outcome in 13q31-amplified cases that had lower expression of these miRNAs [117].

## 5. Malignant Rhabdoid Tumour (MRT)

### 5.1. Histogenesis and Genetics

Malignant rhabdoid tumour is a remarkably aggressive cancer, preferentially occurring in children under three years of age. Depending on the anatomic location where MRT arises, it is variably known as malignant rhabdoid tumor of the kidney, atypical teratoid rhabdoid tumor (when it arises in the central nervous system), or extracranial, extrarenal rhabdoid tumor, when it arises in soft tissues other than these visceral locations. These are however a genetically homogeneous group of tumours sharing the underlying biallelic inactivation of *SMARCB1*, which encodes the SMARCB1/hSNF5/BAF47 subunit of the SWI/SNF chromatin remodelling complex [118,119,120]. The cell of origin of MRT is unknown and suggestions include a primitive mesenchymal cell or a primitive neuroectodermal cell with different histogenetic mechanisms proffered for AT/RT and MRTK. In light of this, it is interesting and revealing to note that miRNA profiling did not show significant differences between AT/RT and MRTK. Gene expression profiling, on the other hand, did show distinct differences between these two entities, but clearly this was not secondary to miRNAs differentially dysregulating gene expression [121]. The gene expression differences related to genes important in brain and renal development, suggesting that the microenvironment within which tumours arose was critical to ultimate gene expression.

### 5.2. Rhabdoid Tumour and Rhabdoid Phenotype

Histologically, MRTs share common features, but which may additionally be observed in a variety of other malignancies, where they are considered to represent a rhabdoid “phenotype”. One such tumour is proximal type epithelioid sarcoma (PES), an aggressive sarcoma of the proximal limbs/limb girdle area, occurring mainly in young adults. Its counterpart, conventional epithelioid sarcoma (CES), a more indolent peripheral sarcoma often affecting somewhat older patients, lacks the rhabdoid phenotype. While all of these tumours (MRT, PES and CES) generally show a lack of INI-1 immunoreactivity, reports of the incidence of underlying genetic inactivating mutations vary greatly. In MRT, complete inactivation of SMARCB1 (or rarely SMARCA4) is integral to the diagnosis. In a study of miRNA profiling differences between MRT, CES and PES, Kohashi *et al.* found that CES and PES clustered together while MRT formed a distinct set [122]. Differential expression of *miR-*193-5p was diametrically linked to SMARCB1 mutation, insofar as CES or PES without SMARCB1 inactivation showed high *miR-*193-5p levels which could provide an alternative mechanism for SMARCB1 inactivation (Table 1).

### 5.3. AT/RT and Copy Number Decrease of Let7a/Let7b; Over-Expression of miR221, 222

Whole exome DNA sequencing of an adult AT/RT revealed a copy number decrease at the let7a/let7b miRNA locus [123]. It was of particular interest then to note that a majority of further AT/RTs analysed showed high levels of HMGA2, a purported target of let7a/7b and a known oncoprotein [124,125]. In this vein, let-7 over-expression or HMGA2 knock-down both led to suppression of *in vitro* growth of G401 rhabdoid tumor cells [123], although the authors admit that HMGA2 is only one potential target of let 7a/7b and the study was focused on this axis only.

A further study comparing miRNA profiles of AT/RT, medulloblastoma and normal brain tissue found relative over-expression of miRNA-221 and 222 in AT/RT [126]. They investigated p27^Kip1^ (CDKN1B), a known target of miRNA-221 and -222 and demonstrated low expression thereof, suggesting potential targeting of p27^Kip1^ by these miRNAs. This report was limited to these selected observations only [126] (Table 1).

## 6. Synovial Sarcoma (SS)

### 6.1. Clinical Background, Tumour Genetics and Epigenetic Modifications

Synovial sarcoma (SS) is a high-grade malignancy and accounts for 5%–10% of soft tissue sarcomas, mainly developing in the juxta-articular soft tissues of adolescents and young adults. Combination therapies have improved the survival rate but it remains very poor for metastatic disease. SS is characterised by chromosomal translocation between chromosomes X and 18 resulting in one of three fusion oncogenes—SS18-SSX1, SS18-SSX2 or SS18-SSX4. The translocation is present in >95% of cases [127]. It has recently emerged that this fusion product evicts SMARCB1/hSNF5/BAF47 from the SWI/SNF chromatin remodelling complex along with wild-type SS18 [128] and that the modified complex then targets the SOX2 locus, inducing expression thereof which leads to increased cell proliferation, contributing to oncogenesis, so chromatin-related changes may ultimately hold the key, to a great extent, to the development of synovial and indeed other sarcomas; however, further epigenetic modifications, including through miRNAs, may also play a role.

### 6.2. miRNAs Differentially Expressed in SS Compared with Other Soft Tissue Sarcomas

Comparing the miRNA profiles of 76 soft tissue sarcomas comprising eight subtypes—SS, leiomyosarcoma (LMS), malignant peripheral nerve sheet tumors (MPNST), undifferentiated high-grade pleomorphic sarcoma (UPS), myxoid fibrosarcoma (MFS), pleomorphic liposarcoma (PLS), dedifferentiated liposarcoma (DDLS) and myxoid liposarcoma (MLS)—25 miRNAs were differentially expressed in SS, including 14 over-expressed and 11 under-expressed compared to the other sarcoma subtypes Renner *et al.* [129], suggesting that these tumours can be accurately classed according to their miRNA profiles. Most members of the *miR-*200 family (*miR-*200a, *miR-*200b and *miR-*429) and *miR-*183 family (*miR-*182, *miR-*183 and *miR-*96) were found to be consistently over-expressed in SS. Increased levels of *miR-*200a/b and *miR-*429 were detected in the SS cell lines HS-SY-II and 1273 but not in SYO-1, Fuji and CME cells. RT-qPCR confirmed significantly higher expression of *miR-*200a/b/c and *miR-*429 in SS compared to other STS [129].

miRNA profiling of 10 SS, five Ewing sarcomas and five normal muscle samples revealed 35 miRNAs significantly differentially expressed in SS (21 up-regulated and 14 down-regulated), with some of the up-regulated miRNAs transcribed from the same chromosomal regions, including let-7e, *miR-*99b and *miR-*125a-3p from 19q13.4 and *miR-*376a, *miR-*381 and *miR-*376c from 14q32.3 [130]. No significantly differentially expressed miRNAs localised to the chromosomal breakpoints in either sarcoma. Skeletal muscle-specific miRNAs *miR-*1, *miR-*133 and *miR-*206 were found to be down-regulated relative to skeletal muscle in both sarcoma types. In SS cell line HS-SY-II, inhibition of let-7e and *miR-*99b significantly reduced cell proliferation compared to controls. Target prediction algorithms suggest both HMGA2 and SMARCA5 are potential targets of *miR-*99b and let-7e and indeed, following silencing of *miR-*99b and let-7e, RT-PCR and Western blotting showed increased expression of both HMGA2 and SMARCA5. These findings suggest a role whereby these dysregulated miRNAs contribute to oncogenesis and raise consideration for targeting them therapeutically (Table 1).

### 6.3. miR-17; miR-183 Potentially Useful Therapeutic Targets

A further promising miRNA target was presented in the work of Minami *et al.* [131]. In this study, a cancer-related miRNA screen was performed using the OncomiR miRNA Precursor Virus Library system, which contains 140 cancer miRNAs. The vector was transfected into SS cell lines Fuji and HS-SYII and colony formation was examined in soft agar. Infected Fuji cells, but not HS-SYII cells, formed numerous large colonies compared to uninfected controls [131]. RT-qPCR using the OncomiR miRNA Precursor Virus Library primer identified high expression of *miR-*17-5p, a member of the *miR-*17-92 cluster. Forced expression of SS18-SSX2, but not SS18-SSX1, promoted the endogenous expression of *miR-*17 in Fuji cells, whereas depletion of endogenous SS18-SSX2 by siRNA reduced expression levels of *miR-*17 in a concentration-dependent manner, indicating SS18-SSX2 is able to regulate *miR-*17 expression. A luciferase reporter vector showed *miR-*17 targets p21, and reduction in p21 protein levels was confirmed by Western blot. An additional noteworthy finding in this study was that marked p21 expression evoked by doxorubicin treatment was also reduced by *miR-*17 over-expression. Forced expression of *miR-*17 was able to restore doxorubicin-induced growth suppression in Fuji cells. Inhibition of *miR-*17 was found to restore p21 protein levels and reduce cell growth [131] (Table 1).

Sarver *et al.* focused on *miR-*183 (part of an evolutionary conserved cluster containing *miR-*183, *miR-*96 and *miR-*182) which emerged as showing over-expression on network analysis of sarcomas, especially SS but also the different types of RMS [132]. By luciferase assay in Fuji and SYO-1 cells, they showed that *miR-*183 binds EGR1, which has been previously shown to be decreased in SS [133]. Knockdown of *miR-*183 resulted in increased levels of *EGR1* mRNA and protein levels. Following knockdown of *miR-*183, PTEN was also found to be up-regulated. Global transcriptome analysis revealed that knockdown of *miR-*183 was able to modulate the transcriptional profiles of tumour cell lines *in vitro*. mRNA profiling of SS and RMS tumour samples showed that mRNAs that exhibit positive and negative correlations with *miR-*183 are conserved in primary tumours. Inhibition of *miR-*183 was found to decrease cell migration and EGR1 knockdown could functionally restore the anti-*miR-*183 migration phenotype [132] (Table 1).

**Table 1:** MiRNAs Dysregulated in Pediatric Sarcomas.The table lists miRNAs described in the literature as showing dysregulated expression in the various pediatric sarcomas together with expression change observed, cellular effect and target gene(s) where known, as well as reference where the findings are cited.

Tumor Type	miRNA	Expression	Function	Target	Ref.
Osteosarcoma	*miR-*34	Reduced expression reported in Osteosarcoma	Promotes cell growth arrest and cell death		[54]
	*miR-*31		Over-expression inhibits proliferation in Osteosarcoma cell lines, while loss of *miR-*31 causes defects in the TP53 pathway	E2F2	[55]
	*miR-*215	Induced by TP53, tumour supressor	Reduces osteosarcoma cell colony formation, resistance to methotrexate	p21	[56,68]
	*miR-*192	Induced by TP53	Reduces cell proliferation	p21 expression	[56]
	*miR-*223	Reduced expression reported in Osteosarcoma	Low *miR-*223 and high ECT2 associated with tumour grade, chemo-resistance, metastasis and recurrence	ECT2	[57]
	*miR-*183		Low *miR-*183 and high EZRIN associated with tumour grade, chemo-resistance, metastasis and recurrence	EZRIN	[57]
	*miR-*199a-3p		Inhibits cell growth and migration	MET, mTOR and STAT3	[58]
	*miR-*125b		Functions in a feedback loop with STAT3, suppresses cell proliferation and migration	STAT3	[59]
	*miR-*133a/b	Significant down-regulation reported in Osteosarcoma	Over-expression of *miR-*133b reduces cell proliferation, migration and invasion, promotes apoptosis	Predicted target pathways include: Bcl2L2, Mcl1, IGF1R, MET, pAKT, PTEN and FAK	[60]
	*miR-*20a		Increased *miR-*20a expression combined with decreased Fas expression associated with reduced FasL-induced apoptosis and cell cytotoxicity and contributes to the development of lung metastases		[113]
	miRNAs at 14q32 locus	Down-regulated in Osteosarcoma	Subset of miRNAs from this cluster act cooperatively to down-regulate MYC, which in turn down-regulates the *miR-*17-92 cluster	MYC	[62]
	*miR-*214		Increased expression predicitive of a poor pre-operarive response to chemotherapy, shorter OS and EFS in high expressing tumours		[63]
	*miR-*210		Increased expression predicitive of a poor pre-operarive response to chemotherapy, shorter OS and EFS and increased metastaitc risk in high expressing tumours		[64]
	*miR-*27a		Higher in cases that developed metastasis		[65]
	*miR-*181c		Higher in cases that developed metastasis		[65]
	*miR-*451		Higher expression correlates with a better chemotherapy response		[65]
	*miR-*15b, *miR-*16		High expression may contribute to an improved response to chemotherapy	Bcl2	[65]
	*miR-*143	Down-regulated in Osteosarcoma	Tumour Suppressor in Osteosarcoma	Bcl2	[66]
	*miR-*33a	Higher expression in chemo-resistant Osteosarcoma	High *miR-*33a and low TWIST expression identified in cisplatin resistant tumours	TWIST	[67]
	*miR-*140	Increased expression in chemo-resistant Osteosarcoma	Associated with resistance to doxorubicin, cisplatin and ifosamide		[68]
	*miR-*92a, *miR-*99b, *miR-*193a-5p, *miR-*422a	Part of a 5-miRNA signature, over-expressed in Osteosarcoma	Associated with ifosamide resistance	TGFb, Wnt, MAPK pathway	[69]
	*miR-*132	Part of a 5-miRNA signature, under-expressed in Osteosarcoma	Associated with ifosamide resistance	TGFb, Wnt, MAPK pathway	[69]
Ewing Sarcoma (ES)	*miR-*17-92 cluster, *miR-*106b-25 cluster, *miR-*106a-363 cluster	Up-regulated in ES	members of each cluster are associated with poor prognosis, blockade of *miR-*106a-363 inhibits colony formation and is associated with *miR-*15a over-expression		[78,79]
	let-7a		Growth/tumour suppressor miRNA, EWSR1-FLI1 binds the let-7a promoter and represses formation of the mature miRNA, over-expression reduces cell growth, migration and invasion	EWSR1-Fli1, HMGA2, IGF2BP1, Lin28	[80,81]
	*miR-*100	Up-regulated following EWSR1-Fli1 silencing	Growth suppressive in ES	IGF1R, mTOR	[82]
	*miR-*27a	Up-regulated following EWSR1-Fli1 silencing	Growth suppressive in ES	IGF1a	[82]
	*miR-*30a-5p		Over-expression reduces cell proliferation and invasion	CD99	[83]
	*miR-*145	Low/absent expression in ES	Directly binds Fli1 3′ UTR, acts in a mutually repressive feedback loop with EWSR1-Fli1	EWSR1-Fli1, OCT4, SOX2	[89,90]
	*miR-*34a, *miR-*23a, *miR-*92 *miR-*490-3p, *miR-*130b		Part of a miRNA signature associated with survival predicition as independent predictors of outcome		[79]
	*miR-*106b *miR-*93 *miR-*181b *miR-*101 *miR-*30b	Over-expression in ES compared to mesenchymal stem cells			[92]
	*miR-*193a-3p, *miR-*100 *miR-*22 *miR-*21 *miR-*574-3p	Over-expression in ES compared to mesenchymal stem cells			[92]
	*miR-*708	Reduced expression in ES	Increased expression of *miR-*708 sensitized ES cells to etoposide	EYA3	[98]
	*miR-*125b		Highly expressed in doxorubicin-resistant cell lines, silencing of *miR-*125b enhances cell death by increased sensitivity to doxorubicin, vincristine, etoposide and mafosfamide	p53, Bak, PIK3CD	[99]
	*miR-*22		Over expression in ES cells inhibits colony formation	KDM3A	[86]
Rhabdomyosarcoma (RMS)	*miR-*1 *miR-*133a *miR-*133b *miR-*206	Collectively known as myomiRs and are all found significantly lower in RMS	*miR-*206 is inversely correlated with overall survival. Found to be useful as serum biomarkers for RMS Ectopic expression inhibits cell growth through G1-S pahse arrest and reduces colony formation. Re-expression promotes differentiaiton in RMS cells	HDAC4, PAX3, BAF53	[103,105,106,107,108,110]
	*miR-*203	Down-regulated in RMS	Down-regulation casued by promoter hypermethylation and *miR-*203 expression can be reactivated by 5-aza-dC. Re-expression promotes terminal myogenic differentiaiton	p63 (which in turn p63 targets JAGGED in the Notch pathway and LIFR in the JAK/STAT pathway)	[109]
	*miR-*17-92 cluster	Amplification of 13q31 region isassociated with increased expression of 5 miRNAs from this cluster	Significantly worse outcome associated with increased expression of miRNAs within this cluster in 13q31-amplified cases		[117]
	*miR-*450b-3p	Tumour suppressor		TGFβ1, ENOX, PAX9	[114]
	*miR-*29	Epigenetic silencing in RMS	*miR-*29 is silenced through activation of the NFκB-YY1 pathway and myogenic differentiation cannot progress	YY1	[115,116]
	*miR-*26a	Down-regulated in RMS	Involved in Myoblast Differentiation	Targets Ezh2, polycomb group protein	[111]
	*miR-*214	Down-regulated in RMS	Involved in Myoblast Differentiation	N-Ras	[113]
Malignant Rhabdoid Tumour (MRT)	*miR-*193-5p		Differential expression diametrically linked to SMARCB1 mutation		[122]
	let-7a/let-7b	Copy number decrease identified in AT/RT	Over-expression of let-7 supresses MRT cell growth	HMGA2	[123]
	*miR-*221/222	Over-expressed in AT/RT		CDKN1B	[126]

## 7. Conclusions

In conclusion, our understanding of the non-coding genome has been increasing rapidly in the past two decades, during which time miRNAs have enjoyed the greatest attention, revealing their importance in developmental processes but also the significance of their dysregulation in cancer development and progression. Here it has been shown that miRNAs may impact the well-characterised genetic pathways known to be important in specific tumour entities, by exerting oncogenic or tumour-suppressive effects, depending on the target gene(s). Their occasional tissue specificity, notably in the context of myomiRs for example, and their association with disease progression afford them value as biomarkers. Increasing evidence supports their value not only as therapy-predictors but also as therapeutic targets themselves. The future will surely bring major developments in the application of this knowledge to benefit cancer patients, including those afflicted with pediatric sarcomas.
